# The determination of the radiochemical purity of Actinium-225 radiopharmaceuticals: a conundrum

**DOI:** 10.1186/s41181-022-00175-y

**Published:** 2022-09-06

**Authors:** Janke Kleynhans, Adriano Duatti

**Affiliations:** 1 Division of Nuclear Medicine, Stellenbosch University, 7505 Cape Town, South Africa; 2 Department of Chemical and Pharmaceutical Sciences, University of Ferrara, 44121 Ferrara, Italy

## To the editor

### Main text

The recent emphasis on actinium-225 radiopharmaceuticals has led to underestimate a serious problem related to the measurement of their radiochemical purity (RCP). The rules of Good Radiopharmacy Practice (GRP) dictate that RCP of an injectable radiopharmaceutical *must* be determined by a suitable analytical method prior to patient administration. RCP is defined as the ’ratio of the activity of a radionuclide in a *stated chemical species* in a material over the total activity of all species containing that radionuclide in this material’ (Gillings et al. 2021). A logical implication of this definition is that RCP for a radiopharmaceutical can be rigorously determined only after establishing its true molecular identity. Since this fundamental characterization cannot be performed routinely in a radiopharmacy with conventional quality control methods, a widely accepted approach is to first isolate and structurally characterize an *authentic* sample of the chemical species supposed to possess the same composition and molecular architecture of the radiopharmaceutical. This fundamental characterization is usually accomplished by applying techniques for structure determination to macroscopic amounts of the authentic sample synthesized using a stable isotope of the radionuclide incorporated into the radiopharmaceutical. Then, the chromatographic profile of the authentic sample can be compared with the profile of the corresponding radiopharmaceutical both collected using High-Performance Liquid Chromatography (HPLC) and/or Instant Thin-layer Chromatography (ITLC) under the same experimental conditions for cross validation (Thakral et al. 2021; Hooijman et al. 2021). When there exists a close matching between these two chromatographic profiles, it is reasonable to assume that the radiopharmaceutical has the same chemical identity of the authentic sample. After this preliminary characterization, to assess RCP of a radiopharmaceutical prepared routinely in a hospital setting in compliance with GRP rules, it is sufficient to check whether its HPLC or TLC chromatogram always includes the peak corresponding to the authentic sample.

The paucity of purified actinium materials combined with the lack of stable isotopes and measurable spectroscopic properties for this element has prevented the isolation and structural investigation of authentic samples of its coordination compounds and made it arduous to determine their chromatographic properties (Deblonde et al. 2021; Kovács 2021). Without the support of these data, it appears difficult to strictly adhere to the rigorous definition of RCP. The issue of RCP becomes more dramatic considering that the chromatographic profiles of actinium-225 complexes can be measured only after decay of the pure α-emitting radionuclide to its γ emitting daughters. Exemplary are the conventional methods to detect the γ emission of francium-221 after reaching the secular equilibrium with the precursor actinium-225 (Hooijman et al. 2021). Although models (Kelly et al. 2021) have been developed to calculate RCP of actinium-225 radiopharmaceuticals from γ decay of francium-221, this approach raises serious questions about the interpretation of chromatographic results since the integrity of actinium-225 radiopharmaceuticals is always challenged by the so-called alpha recoil effect caused by the emission of the α particle (Kozempel et al. 2018). This process can irreversibly disrupt the molecular scaffolding of the coordination complex, thus making the attribution of chromatographic data to a specific radiolabeled species uncertain. Ultimately, the simple ITLC cannot be considered on its own a suitable method for routinely assessing the RCP of actinium-225 compounds since radiolysis with alpha emitters very efficiently produces impurities that are not identified. These uncertainties demonstrate that accurate measurement of RCP for actinium-225 radiopharmaceuticals still constitutes an unsolved problem in radiopharmacy.

## Data Availability

Not applicable.

## Abbreviations

RCP: Radiochemical purity.
ITLC: Instant Thin-layer chromatography.
HPLC: High Performance Liquid Chromatography.
